# Gla-Rich Protein Across the Chronic Kidney Disease Spectrum: Association with Vascular Calcification Burden and CKD-MBD Disturbances

**DOI:** 10.3390/jcm15093374

**Published:** 2026-04-28

**Authors:** Antun Lončarić, Marlena Išek Lončarić, Diana Balenović, Lara Batičić

**Affiliations:** 1 Department of Cardiology, General Hospital Dr. Ivo Pedišić Sisak, 44000 Sisak, Croatia; antun.loncaric@uniri.hr (A.L.); diana.balenovic@gmail.com (D.B.); 2 Department of Radiology, General Hospital Dr. Ivo Pedišić Sisak, 44000 Sisak, Croatia; marlenaisekloncaric@gmail.com; 3 Department of Medical Chemistry, Biochemistry and Clinical Chemistry, Faculty of Medicine, University of Rijeka, 51000 Rijeka, Croatia

**Keywords:** fibroblast growth factor-23, Gla-rich protein (GRP), Kauppila score, klotho proteins, pulse wave velocity, renal insufficiency, chronic, vascular calcification, vitamin K-dependent proteins

## Abstract

**Background/Objectives**: Vascular calcification and arterial stiffness are common in chronic kidney disease (CKD). Gla-rich protein (GRP) is a vitamin K-dependent protein implicated in mineral biology, but clinical evidence across CKD stages is limited. We evaluated associations of serum GRP with vascular calcification (VC) burden and arterial stiffness across CKD stages, including hemodialysis, compared with controls. **Methods**: In this prospective observational study, 185 adults were enrolled: controls (n = 61), individuals with CKD stage IIIb–IV (n = 61), and individuals with CKD stage V on hemodialysis (HD) (n = 63). Abdominal aortic calcification was assessed by the Kauppila score, and arterial stiffness was assessed by oscillometric pulse wave velocity (PWV). Serum GRP, FGF-23, and β-Klotho (KLb) were measured by ELISA. Non-parametric group comparisons and Bonferroni-corrected Spearman correlations were used. **Results**: GRP differed across groups (*p* < 0.001), showing a non-linear pattern with the lowest values in CKD IIIb–IV. PWV and Kauppila score increased across CKD stages (both *p* < 0.001). After Bonferroni correction, GRP correlated with KLb (ρ = 0.720) and FGF-23 (ρ = 0.625), but not with PWV or Kauppila score. In multivariable analyses, GRP showed a statistically significant but modest association with PWV and Kauppila score. **Conclusions**: In this CKD spectrum cohort, serum GRP was associated with CKD-MBD biochemical markers (KLb and FGF-23) much more strongly than with vascular phenotypes; its associations with vascular calcification burden and arterial stiffness were modest in multivariable modelling, supporting GRP as a marker of the CKD-MBD biochemical profile rather than a strong surrogate of vascular phenotype.

## 1. Introduction

Chronic kidney disease (CKD) represents a major global health burden and is strongly associated with increased cardiovascular morbidity and mortality. Cardiovascular disease (CVD) remains the leading cause of death in patients with CKD, exceeding the risk related to progression to end-stage renal disease (ESRD) itself [1,2]. Among cardiovascular complications, vascular calcification (VC) plays a central role by contributing to arterial stiffness, left ventricular hypertrophy (LVH), impaired coronary perfusion, and adverse cardiovascular outcomes [3,4]. The prevalence and severity of VC increase progressively with declining renal function and are particularly pronounced in advanced stages of CKD and in patients undergoing maintenance hemodialysis [5].

VC in CKD is no longer considered a passive process of calcium–phosphate deposition but rather an actively regulated, cell-mediated phenomenon resembling osteogenic differentiation of vascular smooth muscle cells (VSMCs) [5,6,7]. Disturbances in mineral metabolism, chronic inflammation, oxidative stress, and reduced activity of endogenous calcification inhibitors contribute synergistically to this process [5,7]. In CKD, these processes are tightly linked to chronic kidney disease–mineral and bone disorder (CKD-MBD), a systemic disturbance of mineral metabolism that promotes VC and adverse cardiovascular outcomes. Despite advances in imaging techniques and cardiovascular risk assessment, reliable circulating biomarkers that reflect VC burden and its underlying pathophysiology remain limited. Identification of such biomarkers could facilitate earlier detection and improved cardiovascular risk stratification in CKD patients.

Vitamin K-dependent proteins (VKDPs) play a crucial role in the regulation of tissue mineralization. Matrix Gla protein (MGP) is the most extensively studied inhibitor of VC [6]. More recently, attention has been directed toward Gla-rich protein (GRP), a relatively novel VKDP with unique structural and functional properties [8,9]. GRP contains a high proportion of γ-carboxyglutamic acid (Gla) residues, conferring strong calcium-binding capacity [8,9]. Similar to other VKDPs, its biological activity depends on vitamin K-dependent γ-carboxylation. Undercarboxylated GRP (ucGRP) forms are considered functionally impaired and have been associated with pathological calcification in various tissues [10,11]. Experimental and translational studies indicate that GRP acts as an inhibitor of ectopic calcification by modulating mineral deposition and influencing calciprotein particle (CPP) and extracellular vesicle (EV) biology, both recognized as important mediators of vascular calcification in CKD [11,12]. In addition, GRP appears to exert anti-inflammatory effects in monocytes and macrophages, partly independent of its carboxylation status, linking mineral dysregulation and inflammation—two key mechanisms of CKD-associated vascular disease [13,14].

Clinical data on GRP in CKD remain limited. Available evidence suggests that circulating GRP levels vary across CKD stages and may be associated with VC and arterial stiffness markers [15,16,17]. However, prior studies involved relatively small or selected cohorts and provided limited integration of circulating GRP with clinically applicable measures of VC. Moreover, comparisons across CKD stages and with non-CKD populations remain scarce [15]. There is also limited evidence integrating GRP measurements with imaging-based assessment of VC, such as abdominal aortic calcification scoring, and functional measures of arterial stiffness [18,19]. Clarifying these relationships may help define the role of GRP within the complex network of mineral metabolism disturbances and cardiovascular risk in CKD.

Therefore, the aim of the present study was to evaluate the association between serum GRP levels and VC burden across different stages of CKD, including patients receiving maintenance hemodialysis, and to compare these findings with a control population without CKD. We hypothesized that lower circulating GRP levels would be associated with greater VC burden and increased arterial stiffness, supporting a potential role of GRP as a clinically relevant biomarker of vascular pathology in CKD.

## 2. Materials and Methods

### 2.1. Study Design and Population

This prospective observational study was conducted at the General Hospital Dr. Ivo Pedišić Sisak in collaboration with the University of Rijeka Faculty of Medicine. Adult participants were consecutively recruited between December 2022 and September 2023. Participants were allocated into three groups: patients with chronic kidney disease (CKD) stages IIIb–IV, patients with ESRD receiving maintenance hemodialysis (CKD stage V), and a control group without CKD. CKD staging was based on estimated glomerular filtration rate (eGFR), calculated using the CKD-EPI equation.

Adult participants aged between 18 and 85 years with CKD stages IIIb–IV or receiving maintenance hemodialysis were eligible for inclusion. All participants provided written informed consent prior to enrollment. Patients were excluded if they were younger than 18 or older than 85 years, pregnant or breastfeeding, had previously undergone kidney transplantation, had active malignant disease, chronic liver disease, or disorders of mineral and bone metabolism unrelated to CKD.

The control group consisted of participants without CKD stage III or higher (eGFR ≥ 60 mL/min/1.73 m^2^) and without diagnosed VC or established atherosclerotic CVD, recruited among individuals evaluated for non-cardiovascular conditions. Control participants had no history of coronary artery disease, peripheral arterial disease, myocardial infarction, stroke, or prior coronary or peripheral revascularization procedures. Individuals with CKD stage III or more (eGFR < 60 mL/min/1.73 m^2^), chronic systemic diseases affecting vascular status, disorders of mineral and bone metabolism, active malignancy, or those receiving medications affecting bone metabolism were excluded. Controls were selected to be free of known atherosclerotic CVD and advanced CKD to provide a reference group with minimal overt vascular disease; however, this approach may limit generalizability and accentuate between-group differences. Participants unwilling or unable to provide informed consent were also excluded.

### 2.2. Ethical Approval

The study protocol was approved by the Ethics Committee of the University of Rijeka Faculty of Medicine and by the Ethics Committee of the General Hospital Dr. Ivo Pedišić Sisak. The study was conducted in accordance with the Declaration of Helsinki, and all participants provided written informed consent.

### 2.3. Clinical Assessment

Baseline demographic data, comorbidities, and medication use were recorded at study inclusion. Blood pressure measurements were obtained after at least five minutes of rest in a seated position, and mean values of repeated measurements were used for analysis.

### 2.4. Laboratory Measurements

Venous blood samples were collected at baseline. Routine laboratory analyses were performed immediately, while serum samples intended for biomarker analysis were centrifuged, aliquoted, and stored until further processing. Blood samples from hemodialysis patients were collected immediately before the dialysis session (pre-dialysis). All samples were processed promptly, centrifuged and aliquoted, and stored at −20 °C for up to one month at the local hospital before transfer to the Faculty of Medicine, where they were stored at −80 °C until batch analysis. Freeze–thaw cycles were minimized. Serum concentrations of total GRP (tGRP), FGF-23, and KLb were measured using commercially available enzyme-linked immunosorbent assay (ELISA) kits according to the manufacturers’ instructions: Human Gla-rich protein (GRP) ELISA Kit (Cat. No. E6794Hu; BT Lab/Bioassay Technology Laboratory, Shanghai, China), Human Fibroblast Growth Factor-23 (FGF-23) ELISA Kit (Cat. No. E0059Hu; BT Lab/Bioassay Technology Laboratory, Shanghai, China), and Human Klotho beta (KLb) ELISA kit (Cat. No. E2782Hu; BT Lab/Bioassay Technology Laboratory, Shanghai, China). The GRP assay quantifies total circulating GRP and does not differentiate between carboxylated (cGRP) and ucGRP fractions; therefore, analyses in this study evaluate associations of tGRP with vascular phenotypes and CKD-MBD biomarkers. Laboratory personnel were blinded to clinical and imaging data.

### 2.5. Assessment of Vascular Calcification

Vascular calcification was assessed at baseline using lateral lumbar spine radiographs visualizing the abdominal aorta. Calcification burden was quantified using the validated Kauppila abdominal aortic calcification score (range of 0–24) [18]. Two independent evaluators, blinded to clinical and laboratory data, assessed all radiographs, and disagreements were resolved by consensus.

### 2.6. Assessment of Arterial Stiffness

Arterial stiffness was assessed at baseline noninvasively by oscillometric measurement of pulse wave velocity (PWV) and central hemodynamic parameters using a commercially available device (Agedio B900, IEM GmbH, Stolberg, Germany). Measurements were performed after a standardized resting period in the supine position. Three consecutive measurements were obtained at 5-min intervals, and the mean value was used for analysis.

### 2.7. Echocardiographic Assessment

Transthoracic echocardiography was performed using a commercially available ultrasound system (Vivid S6, GE Vingmed Ultrasound AS, Horten, Norway). Examinations were conducted by experienced cardiologists as targeted echocardiographic assessments focusing on structural parameters relevant to the study protocol. Left ventricular dimensions and wall thickness were obtained from standard parasternal views. In addition, the degree of aortic valve calcification was assessed semiquantitatively using a visual grading scale from 0 to 4, according to previously described methodology [20]. Briefly, a score of 1 indicated partial calcification of a single cusp, a score of 2 indicated partial calcifications of two cusps, a score of 3 indicated extended calcifications of two cusps, and a score of 4 indicated extended calcifications involving all three cusps. Although Doppler measurements of transvalvular velocities and gradients were obtained during routine examinations, these parameters were not included in the present analysis because left ventricular systolic function was not systematically assessed in all participants.

### 2.8. Follow-Up and Clinical Outcomes

Participants were followed for up to 24 months after inclusion. Survival and cardiovascular events were collected from medical records and follow-up contacts. The primary outcome was all-cause mortality and secondary outcomes included MACE. Follow-up was limited to clinical outcomes; vascular phenotype assessments (Kauppila score and PWV) were performed at baseline and were not repeated.

### 2.9. Statistical Analysis

Statistical analyses were performed using IBM^®^ SPSS^®^ Statistics, version 25 (IBM Corp., Armonk, NY, USA). Normality of continuous variables was assessed using the Kolmogorov–Smirnov test. As most variables were non-normally distributed, continuous data are presented as median (interquartile range, IQR), while categorical data are presented as counts and percentages. All tests were two-sided. A *p* value < 0.05 was considered statistically significant. For predefined analyses involving multiple comparisons, Bonferroni correction was applied within each family of tests.

Between-group comparisons across the three study groups (controls, CKD stage IIIb–IV, and CKD stage V on maintenance hemodialysis) were performed using the Kruskal–Wallis test for continuous variables and the chi-square test (or Fisher’s exact test when appropriate) for categorical variables. When an overall group effect was observed, post hoc pairwise comparisons were conducted using Mann–Whitney U tests for continuous variables and z-tests for proportions for categorical variables, with Bonferroni correction.

Associations between GRP and continuous/ordinal study variables (including renal function, CKD-MBD markers, and vascular phenotype measures such as Kauppila score, PWV, and aortic valve calcification score) were assessed using Spearman’s rank correlation coefficient with Bonferroni adjustment.

Multiple linear regression analyses were performed to evaluate independent predictors of vascular calcification burden and arterial stiffness. Separate multiple linear regression models were fitted with Kauppila score and PWV as dependent variables. Both models included age, diabetes, and eGFR as prespecified covariates, and GRP was additionally entered as a forced-entry predictor to evaluate its independent association with each vascular phenotype. Regression assumptions were assessed using standard diagnostic procedures, including collinearity diagnostics, inspection of residual distributions, and evaluation of residual plots for linearity and homoscedasticity. Regression results are reported as unstandardized (B) and standardized (β) coefficients with corresponding *p* values.

For follow-up analyses, exploratory differences in GRP concentrations according to the occurrence of clinical events (0/1) during follow-up were assessed using the Mann–Whitney U test due to non-normal distribution and small subgroup sizes.

## 3. Results

### 3.1. Study Population and Group Distribution

A total of 185 participants were included in the analysis and categorized into three groups according to kidney disease stage: controls (n = 61), CKD stage IIIb–IV (n = 61), and CKD stage V on hemodialysis (HD) (n = 63). Baseline categorical characteristics differed significantly across groups for several clinically relevant variables (Table 1). Compared with controls, CKD groups had a higher prevalence of arterial hypertension, diabetes mellitus, hyperlipidemia, atrial fibrillation and hyperparathyroidism, while sex distribution did not differ significantly between groups. A history of cardiovascular events/procedures (prior MI, PCI, CABG, and stroke) and warfarin therapy were more frequent in CKD groups. During follow-up, all-cause mortality was highest in the HD group (Table 1).

### 3.2. Distribution of Continuous Variables Across CKD Stages

Most continuous variables did not follow a normal distribution; therefore, non-parametric methods were used for group comparisons (Supplementary Table S1).

PWV and Kauppila score were assessed at baseline and analyzed as vascular phenotype measures, whereas follow-up was used only for clinical outcomes.

Kruskal–Wallis analysis showed significant between-group differences in multiple variables reflecting renal function, vascular remodeling/calcification, and arterial stiffness (Table 2). Age differed across groups, with the CKD IIIb–IV group being older than controls and the HD group. Renal function declined progressively across groups, with median eGFR values showing the expected stepwise reduction from controls to CKD IIIb–IV and HD (*p* < 0.001).

Markers of structural cardiovascular remodeling and vascular calcification also differed significantly across groups. Compared with controls, CKD groups had higher values of IVS, LVPW, aortic (AO) score, and Kauppila score, with the highest burden generally observed in advanced CKD/HD. PWV increased progressively across disease stages (controls < CKD IIIb–IV < HD; *p* < 0.001), indicating greater arterial stiffness with CKD progression.

Among mineral metabolism markers, phosphate (P) and PTH were significantly higher in the HD group, while calcium (Ca) was lower in HD. KLb and FGF-23 also differed significantly between groups (both *p* < 0.001), consistent with progressive CKD-MBD dysregulation.

**Figure 1:**
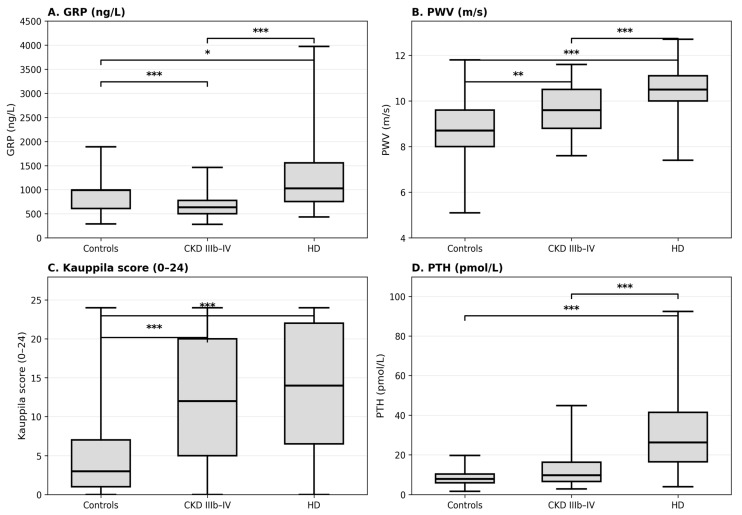
Distribution of vascular and CKD–MBD-related markers across study groups. Box-and-whisker plots show (**A**) GRP (ng/L), (**B**) PWV (m/s), (**C**) Kauppila score (0–24), and (**D**) PTH (pmol/L) in controls, CKD stage IIIb–IV, and hemodialysis (HD) patients. Boxes indicate the interquartile range (IQR) with the median, and whiskers indicate the minimum-to-maximum range. Between-group differences were assessed using the Kruskal–Wallis test, followed by post hoc pairwise Mann–Whitney U tests with Bonferroni adjustment. Significant post hoc differences are indicated by brackets. Asterisks denote Bonferroni-adjusted significance levels: * *p* < 0.05, ** *p* < 0.01, *** *p* < 0.001. Detailed numerical results are provided in Table 2.

Importantly, tGRP levels differed significantly across groups (Kruskal–Wallis χ^2^ = 42.10, *p* < 0.001). Median GRP values were lowest in the CKD IIIb–IV group and higher in both controls and the HD group, suggesting a non-linear relationship between GRP and CKD stage (Table 2, Figure 1).

### 3.3. Correlation Analysis

Although PWV and calcification scores increased significantly across CKD stages at the group level, correlation analysis was performed to determine whether tGRP was directly associated with these vascular phenotypic markers at the individual level or instead more closely linked to CKD-MBD-related biochemical disturbances. Spearman correlation analysis demonstrated the expected overall pattern connecting declining renal function with increasing vascular calcification and arterial stiffness (Supplementary Table S2). To maintain readability, the full correlation matrix is presented in the Supplementary Materials, while the main text highlights the correlations involving tGRP, the central biomarker of this study.

Using a Bonferroni-corrected significance threshold, tGRP showed a negative correlation with eGFR (ρ = −0.220) and positive correlations with KLb (ρ = 0.720), FGF-23 (ρ = 0.625), and phosphate (P) (ρ = 0.241). In contrast, tGRP was not significantly correlated with PWV (ρ = 0.149), Kauppila score (ρ = 0.064), AO score (ρ = 0.100), PTH (ρ = 0.185), calcium (Ca) (ρ = −0.209), or age (ρ = −0.059) after Bonferroni correction.

These findings suggest that, in this cohort, tGRP was more closely associated with CKD-MBD-related biochemical disturbances than with vascular stiffness or calcification burden in pairwise (unadjusted) correlation analysis.

### 3.4. tGRP, Warfarin Therapy, and Clinical Events During Follow-Up

To further assess the potential clinical relevance of tGRP beyond group-wise differences and correlation patterns, we examined whether circulating tGRP levels differed according to clinical events occurring during the follow-up period, as well as according to warfarin use (Table 3).

Using Mann–Whitney U tests, no statistically significant differences in tGRP levels were observed between participants with and without myocardial infarction (MI), PCI, CABG, cerebrovascular insult (CVI), or amputation during follow-up (all *p* > 0.05). Likewise, tGRP did not differ significantly according to all-cause death or cardiovascular death during follow-up (all *p* > 0.05).

No significant difference in tGRP levels was observed between participants receiving and not receiving warfarin therapy (*p* = 0.584). Although GRP is a vitamin K-dependent protein, this finding should be interpreted with caution because the present analysis assessed tGRP, rather than functional GRP fractions that may be more directly influenced by vitamin K antagonism.

Overall, these exploratory analyses did not support an association between tGRP and short-term clinical event occurrence during follow-up in the present cohort. However, event-positive subgroups were very small for several comparisons, substantially limiting statistical power; therefore, these negative findings should be interpreted with caution and should not be considered definitive evidence regarding the prognostic utility of tGRP.

### 3.5. Multivariable Predictors of Kauppila Score

To further explore whether tGRP contributed independently to vascular calcification burden beyond established clinical determinants, an additional multiple linear regression analysis was performed with Kauppila score as the dependent variable and age, diabetes, tGRP, and eGFR as independent variables (Table 4). Although tGRP was not significantly associated with Kauppila score in unadjusted correlation analysis, it was included in this model because of its biological relevance to calcification processes.

The regression model was statistically significant (F(4,180) = 36.471, *p* < 0.001) and explained 39.4% of the variance in Kauppila score (R^2^ = 0.394). Older age (B = 0.330, β = 0.372, *p* < 0.001), diabetes (B = 3.808, β = 0.224, *p* < 0.001), higher tGRP (B = 0.002, β = 0.135, *p* = 0.027), and lower eGFR (B = −0.062, β = −0.248, *p* < 0.001) were independently associated with higher Kauppila score. However, the standardized effect size of tGRP was small relative to the other predictors.

These findings suggest that tGRP showed a statistically independent but modest association with abdominal aortic calcification burden after adjustment for age, diabetes, and renal function.

### 3.6. Multivariable Predictors of PWV

To further evaluate whether tGRP contributed independently to arterial stiffness beyond established clinical determinants, a multiple linear regression analysis was performed with PWV as the dependent variable and age, diabetes, tGRP, and eGFR as independent variables (Table 5). Although tGRP was not significantly associated with PWV in unadjusted correlation analysis, it was included in the multivariable model because of its biological relevance and potential role in vascular pathology.

The regression model was statistically significant (F(4,180) = 37.794, *p* < 0.001) and explained 45.6% of the variance in PWV (R^2^ = 0.456). Age (B = 0.064, β = 0.464, *p* < 0.001), tGRP (B = 0.0002, β = 0.140, *p* = 0.015), and eGFR (B = −0.013, β = −0.325, *p* < 0.001) were significant independent predictors of PWV, whereas diabetes was not independently associated with PWV after adjustment (B = 0.281, β = 0.107, *p* = 0.063).

These findings indicate that older age and lower renal function were the main independent predictors of higher arterial stiffness in the present cohort, while tGRP showed a statistically significant but modest independent association with PWV.

### 3.7. tGRP and eGFR as Predictors of KLb and FGF-23

To further examine the relationship of tGRP with CKD-MBD-related biochemical markers, two multiple linear regression analyses were performed with KLb and FGF-23 as dependent variables and eGFR and tGRP as independent variables (Table 6 and Table 7).

For KLb, the regression model was statistically significant (F(2,78) = 204.30, *p* < 0.001) and explained 83.9% of the variance (R^2^ = 0.839). tGRP was a significant independent predictor (B = 0.029, β = 0.904, t = 19.434, *p* < 0.001), whereas eGFR was not independently associated with KLb in this model (B = −0.053, β = −0.057, t = −1.235, *p* = 0.221) (Table 6).

For FGF-23, the regression model was also statistically significant (F(2,80) = 147.028, *p* < 0.001) and explained 78.5% of the variance (R^2^ = 0.785). Again, tGRP remained a significant independent predictor (B = 0.210, β = 0.875, t = 16.558, *p* < 0.001), while eGFR was not independently associated with FGF-23 (B = −0.395, β = −0.062, t = −1.165, *p* = 0.248) (Table 7).

These analyses further support that, in the present cohort, tGRP showed a substantially stronger independent association with KLb and FGF-23 than eGFR in these two-predictor models, consistent with the correlation findings reported in Section 3.3 (Table 6 and Table 7; Supplementary Table S2). However, given the unusually high explained variance, these models should still be interpreted with appropriate caution despite acceptable regression diagnostics.

## 4. Discussion

In this observational cohort including controls, patients with CKD stage IIIb–IV, and patients on maintenance hemodialysis, circulating tGRP levels differed significantly across groups but followed a non-linear pattern, with the lowest values observed in CKD IIIb–IV and higher values in controls and HD patients. In contrast, vascular calcification burden (Kauppila score) and arterial stiffness (PWV) increased progressively across CKD stages, consistent with the expected CKD vascular phenotype and the broader CKD–MBD framework [21,22,23,24]. However, given the substantial between-group differences in cardiovascular risk profiles, groupwise comparisons should be interpreted cautiously and primarily as reflecting the integrated CKD/comorbidity phenotype rather than CKD stage in isolation.

Importantly, GRP is a vitamin K-dependent protein whose calcification-related activity is influenced by γ-carboxylation status; however, this study quantified tGRP using an assay that does not differentiate carboxylated and undercarboxylated GRP fractions. Therefore, the present analyses address the clinical associations of tGRP with vascular phenotypes and CKD–MBD biomarkers rather than the functional GRP proteoforms that may more directly mediate calcification biology.

Notably, most available clinical studies in CKD and dialysis cohorts have likewise quantified tGRP using ELISA-based methods (often reported as total GRP or tGRP), rather than carboxylated/undercarboxylated fractions, which supports the clinical relevance of evaluating tGRP as a pragmatic biomarker while acknowledging the limitation regarding functional status [15,16,17,25].

Despite these clear group-wise differences, tGRP did not show robust associations with PWV, Kauppila score, AO score, or PTH after correction for multiple testing. By contrast, tGRP was associated with eGFR, phosphate, KLb, and FGF-23, and in two-predictor models (eGFR + GRP), tGRP remained the only significant independent predictor of KLb and FGF-23. Importantly, KLb represents β-Klotho (KLB), which primarily acts as an obligatory co-receptor for endocrine FGF19/FGF21 signaling and differs from α-Klotho, the canonical co-receptor for FGF-23; therefore, these associations likely reflect shared CKD-related biochemical alterations rather than direct ligand–co-receptor coupling. Accordingly, the tGRP–KLb association should not be interpreted as evidence of direct FGF-23 pathway coupling; instead, it likely reflects shared CKD-related biochemical milieu (e.g., mineral stress, inflammation, and endocrine/metabolic adaptation), and may also be influenced by CKD stage and/or shared clearance effects in advanced kidney disease. In this context, KLb may primarily represent broader FGF19/FGF21-related biology rather than canonical FGF-23/α-Klotho signaling [26,27].

At the same time, the multivariable analyses add important nuance to the unadjusted findings. Although tGRP was not significantly correlated with Kauppila score or PWV in pairwise analysis after correction for multiple testing, it retained a statistically significant independent association with both outcomes after adjustment for age, diabetes, and eGFR. However, these associations were modest in magnitude, with substantially smaller standardized effect sizes than those observed for age, eGFR, or, in the case of Kauppila score, diabetes. Thus, the present data do not support interpretation of tGRP as a dominant determinant of vascular calcification burden or arterial stiffness; rather, they suggest a weak but detectable independent association that becomes apparent only after accounting for key clinical covariates. Taken together, these findings suggest that, in the present cohort, tGRP was much more strongly associated with the biochemical CKD–MBD profile than with vascular structural and functional phenotypes or short-term outcomes.

The observed non-linear tGRP distribution across CKD stages is an important finding. Several non-mutually exclusive mechanisms may contribute to higher tGRP levels in HD compared with CKD IIIb–IV. Because blood samples in HD patients were collected pre-dialysis, an immediate post-dialysis hemoconcentration effect is unlikely; however, differences in volume status, dialysis-related handling/clearance of circulating proteins, and residual kidney function may still influence measured concentrations. In addition, ESRD is characterized by pronounced mineral stress and altered EV and CPP dynamics, which could plausibly modulate circulating tGRP as part of a compensatory or stress-response pattern [12]. In line with this, recent dialysis data have reported tGRP in dialysis cohorts and its associations with vascular calcification and mineral/inflammatory markers, supporting the concept that GRP may track the mineral-stress/inflammatory milieu in advanced kidney disease [17]. This broader interpretation is consistent with prior discussions highlighting a potential dual role of GRP at the interface of calcification and inflammation in CVD [28]. Finally, residual confounding by dialysis vintage and potential assay/matrix effects in uremic serum cannot be excluded and may partly contribute to the observed pattern. Overall, these observations support stage-specific regulation of tGRP rather than a simple monotonic relationship with declining kidney function.

This interpretation is biologically plausible given mechanistic evidence indicating that GRP participates in calcification inhibition, EV biology, VSMC osteogenic differentiation, and immunomodulatory pathways relevant to VC [11,29]. More broadly, recent reviews of extrahepatic VK-dependent Gla proteins emphasize that functional status and assay comparability are critical when interpreting circulating levels across studies, particularly for proteins implicated in ectopic calcification and cardiometabolic disease [30].

A mechanistic framework that can integrate these observations is that CKD-associated VC is increasingly recognized as a vesicle- and particle-mediated process rather than passive mineral deposition alone. Matrix vesicles represent critical nucleation platforms for calcium–phosphate crystallization and are considered promising therapeutic targets in VC [31].

In CKD, EV (including exosomes) contribute to intercellular communication and can propagate pro-calcific signaling, thereby linking mineral stress to vascular remodeling [32]. Translational data support the importance of circulating mineral stress: calcification propensity (T50) and CPP counts are increased in CKD, and CPP counts are associated with markers of vascular remodeling, suggesting that dynamic circulating determinants contribute to vascular pathology beyond static imaging scores [33]. More recently, nephrology-focused reviews have highlighted circulating small EV as potential mediators of inter-organ communication between the kidney and vasculature, reinforcing their relevance for CKD-associated VC [34]. Together, these concepts provide a plausible explanation for why tGRP may more strongly reflect biochemical CKD–MBD activity (e.g., phosphate-related stress and endocrine signaling) than cumulative calcification burden or stiffness indices at the individual level.

A key implication of our results is that group-level trends and individual-level correlations should not be interpreted as equivalent. CKD progression simultaneously affects renal function, phosphate handling, FGF-23-related signaling, inflammation, blood pressure, and vascular remodeling [21,22,23]. This is also consistent with contemporary overviews linking CKD pathophysiology to accelerated coronary and systemic vascular calcification [35]. Accordingly, a biomarker may differ across CKD stages while failing to show a robust direct association with a specific vascular phenotype once multiplicity is accounted for. At the same time, a modest independent association may still emerge after multivariable adjustment. Consistent with this, age, diabetes, and eGFR independently predicted Kauppila score, while age and eGFR predicted PWV in our multivariable models. In the adjusted models, tGRP also showed a statistically significant but comparatively small independent contribution to both Kauppila score and PWV.

In conclusion, in this mixed CKD cohort, tGRP was more strongly linked to CKD–MBD biochemical disturbances (particularly KLb and FGF-23) than to VC burden, arterial stiffness, or short-term outcomes. Although tGRP showed statistically significant independent associations with Kauppila score and PWV in adjusted models, these effects were modest and should not be overinterpreted as evidence of a major vascular determinant. These findings support a role for tGRP as a marker of the CKD–MBD biochemical phenotype, while further studies using standardized assays and functional GRP fractions are needed to clarify its vascular and prognostic utility.

## 5. Limitations

This study has several limitations. First, the single-center observational design and the moderate sample size limit generalizability, and some subgroup analyses were constrained by small numbers. Biomarkers were measured once at baseline; therefore, longitudinal biomarker dynamics in relation to vascular phenotypes could not be assessed. Vascular phenotype assessments (Kauppila score and PWV) were performed at baseline and were not repeated; therefore, changes in vascular calcification or arterial stiffness over time could not be evaluated.

Second, KLb and FGF-23 were available only in subsets of participants because a subset of stored samples became unusable following an unexpected freezer malfunction with partial thawing, resulting in insufficient or degraded material for reliable ELISA quantification. Therefore, analyses involving KLb and FGF-23 should be interpreted cautiously because selection bias cannot be ruled out and the corresponding subsets may not fully represent the entire cohort. To assess potential selection bias, baseline characteristics of participants with versus without available KLb/FGF-23 measurements were compared (Supplementary Table S3). These comparisons showed significant differences in sex distribution and eGFR, whereas age, diabetes, hypertension, Kauppila score, PWV, and BMI did not differ significantly between participants with and without available KLb/FGF-23 measurements. In addition, the very high R^2^ values observed in the two-predictor models for KLb and FGF-23 should be interpreted cautiously, even though regression diagnostics did not indicate major violations of model assumptions.

Third, only tGRP was measured. Because GRP activity is influenced by vitamin K-dependent γ-carboxylation, we could not assess whether cGRP or ucGRP fractions show stronger vascular associations. In addition, vitamin K status (dietary intake and supplementation) was not systematically captured; therefore, inference regarding GRP γ-carboxylation (functional status) is limited.

Fourth, the control group was intentionally selected to be free of established atherosclerotic CVD and known VC, with preserved renal function (eGFR ≥ 60 mL/min/1.73 m^2^). While this provided a “clean” reference group with minimal overt vascular disease, it may accentuate between-group differences compared with an unselected real-world population and limits generalizability. Moreover, CKD groups differed from controls in multiple cardiovascular risk factors and comorbidities; therefore, between-group differences in GRP should not be interpreted as being attributable to CKD stage alone, but rather as reflecting the broader integrated CKD phenotype and comorbidity burden.

Finally, follow-up analyses comparing tGRP levels according to clinical events were exploratory and underpowered due to the small number of events in subgroups. Accordingly, these results should not be interpreted as definitive evidence regarding the prognostic utility of tGRP. In addition, direct comparison of tGRP concentrations across studies should also be made cautiously because assay-specific calibration, reporting units (e.g., ng/L vs. ng/mL vs. pg/mL), and differences in analyte detection (total vs. functional fractions) may limit comparability [17,29,36].

## 6. Conclusions

In this mixed CKD cohort, tGRP levels differed significantly across CKD stages and followed a non-linear distribution pattern. While vascular calcification burden and arterial stiffness increased progressively with CKD severity, tGRP did not show robust unadjusted associations with vascular phenotypes after correction for multiple testing. However, in adjusted models, tGRP showed statistically significant but modest independent associations with vascular calcification burden and arterial stiffness. Exploratory follow-up analyses were underpowered and did not support a clear relationship between tGRP and short-term clinical events. In contrast, tGRP was much more strongly associated with CKD–MBD-related biochemical markers, particularly KLb and FGF-23. Taken together, these findings suggest that tGRP primarily reflects the biochemical background of CKD–MBD rather than serving as a strong standalone surrogate of vascular calcification burden, arterial stiffness, or short-term cardiovascular risk. This distinction is clinically relevant, as it underscores the need to interpret GRP within the broader context of mineral metabolism rather than as an isolated vascular biomarker.

Future research should focus on the standardization of GRP measurement and the differentiation of its functional fractions, such as carboxylated and undercarboxylated forms, which may have distinct biological activities and stronger pathophysiological links to vascular calcification. Longitudinal studies with extended follow-up, integration of multimodal imaging, and mechanistic investigations are warranted to clarify whether specific GRP isoforms contribute directly to calcification processes or may serve as therapeutic targets within the CKD–MBD axis.

## Figures and Tables

**Table 1:** Baseline categorical characteristics and follow-up outcomes across study groups (controls, CKD IIIb–IV, and HD).

Variable	Controls (n = 61)	CKD IIIb–IV (n = 61)	HD (n = 63)	*p*-Value
Sex (male), n (%)	24 (39.3)	31 (50.8)	36 (57.1)	0.134
Arterial hypertension, n (%)	36 (59.0)	61 (100.0)	61 (96.8)	<0.001
Diabetes mellitus, n (%)	14 (23.0)	35 (57.4)	29 (46.0)	<0.001
Hyperlipidemia, n (%)	27 (44.3)	51 (83.6)	38 (60.3)	<0.001
Atrial fibrillation, n (%)	4 (6.6)	13 (21.3)	18 (28.6)	0.006
Hyperparathyroidism, n (%)	5 (8.2)	30 (49.2)	62 (98.4)	<0.001
Warfarin therapy, n (%)	2 (3.3)	8 (13.1)	19 (30.2)	<0.001
Prior myocardial infarction, n (%)	0 (0.0)	12 (19.7)	12 (19.0)	0.001
Prior PCI, n (%)	0 (0.0)	14 (23.0)	10 (15.9)	0.001
Prior CABG, n (%)	0 (0.0)	2 (3.3)	3 (4.8)	0.248
Prior stroke (CVI), n (%)	0 (0.0)	3 (4.9)	6 (9.5)	0.048
All-cause death during follow-up, n (%)	1 (1.6)	4 (6.6)	9 (14.3)	0.027
Cardiovascular death during follow-up, n (%)	0 (0.0)	1 (1.6)	3 (4.8)	0.179

Abbreviations: CKD, chronic kidney disease; HD, hemodialysis; PCI, percutaneous coronary intervention; CABG, coronary artery bypass grafting; CVI, cerebrovascular insult. Data are presented as n (%). Comparisons were performed using the chi-square test or Fisher’s exact test, as appropriate.

**Table 2:** Continuous variables across study groups (controls, CKD stage IIIb–IV, and hemodialysis).

Variable	Controls	CKD IIIb–IV	HD	χ^2^ (Kruskal–Wallis)	*p*-Value
Age, years	63 (58–65.5)	73 (65.5–76.5)	69 (62–76)	34.84	<0.001
eGFR (mL/min/1.73 m^2^)	83 (74.5–91.5)	31 (22–37)	6 (4–8)	163.12	<0.001
IVS (mm)	11 (10–11)	12 (11–13)	12 (11–14)	34.98	<0.001
LVPW (mm)	10 (9–11)	11 (10–12)	12 (10–13)	34.75	<0.001
AO score	1 (0–2)	2 (1–3)	3 (2–3)	49.33	<0.001
LVDd (mm)	49 (46–52)	51 (47–55)	53 (48–55)	9.28	0.010
Systolic BP (mmHg)	130 (122.5–138.5)	135.37 (128–144.5)	141.85 (134–148)	27.50	<0.001
Diastolic BP (mmHg)	82 (78–87.5)	83 (80–87)	82.49 (77–87)	0.96	0.620
PWV (m/s)	8.7 (7.95–9.6)	9.6 (8.7–10.5)	10.5 (9.9–11.1)	46.41	<0.001
BMI (kg/m^2^)	27.5 (24.9–29.45)	29.6 (26.9–32.25)	27.9 (24.6–33.4)	11.14	<0.001
Kauppila score	3 (1–7.5)	12 (4.5–20.5)	14 (5–23)	41.74	<0.001
KLb (nmol/L)	25.33 (17.9–31.8)	21.47 (17.39–23.74)	30.98 (23.38–68.74)	23.70	<0.001
tGRP (ng/L)	994.93 (607.98–994.93)	638.67 (499.4–781.1)	1028.41 (751.80–1574.07)	42.10	<0.001
FGF-23 (pg/mL)	246.26 (188.82–323.28)	188.25 (161.68–215.79)	264.12 (228.85–444.28)	23.66	<0.001
Ca (mmol/L)	2.35 (2.28–2.41)	2.38 (2.29–2.48)	2.21 (2.11–2.31)	41.89	<0.001
P (mmol/L)	1.23 (1.11–1.34)	1.23 (1.09–1.42)	1.49 (1.22–1.78)	26.85	<0.001
PTH (pmol/L)	7.93 (5.81–10.66)	9.8 (6.17–16.57)	26.27 (16.52–41.72)	72.07	<0.001

Abbreviations: eGFR, estimated glomerular filtration rate; IVS, interventricular septum thickness; LVPW, left ventricular posterior wall thickness; AO score, aortic calcification score; LVDd, left ventricular end-diastolic diameter; BP, blood pressure; PWV, pulse wave velocity; BMI, body mass index; KLb, β-Klotho; tGRP, total Gla-rich protein; PTH, parathyroid hormone. KLb and FGF-23 were available in subsets of participants; sample sizes are provided in Supplementary Table S3. Data are presented as median (IQR). Group comparisons were performed using the Kruskal–Wallis test.

**Table 3:** tGRP levels according to warfarin therapy and clinical events during follow-up.

Event/Condition	No Event/Condition: tGRP, Median (IQR)	Event/Condition Present: tGRP, Median (IQR)	Mann–Whitney U	*p*-Value
Myocardial infarction (MI) during follow-up	798.31 (608.40–1017.22), n = 178	772.97 (492.27–1574.07), n = 7	621.00	0.989
PCI during follow-up	789.24 (608.82–1013.84), n = 179	959.34 (479.51–2402.99), n = 6	470.00	0.603
CABG during follow-up	807.12 (608.40–1027.63), n = 182	638.67 (619.58–660.08), n = 3	159.00	0.215
Stroke/CVI during follow-up	789.24 (607.15–999.31), n = 183	1371.39 (1291.34–1451.45), n = 2	59.00	0.099
Amputation during follow-up	791.13 (608.82–1006.57), n = 181	826.88 (400.65–1459.15), n = 4	342.50	0.854
CV death during follow-up	791.13 (607.98–1020.61), n = 181	874.68 (524.16–1424.65), n = 4	353.00	0.932
All-cause death during follow-up	791.13 (600.49–999.31), n = 171	829.98 (665.79–999.31), n = 14	1061.00	0.480
Warfarin therapy	790.18 (602.16–994.93), n = 156	834.75 (617.98–1250.44), n = 29	2117.00	0.584

Abbreviations: tGRP, total Gla-rich protein; PCI, percutaneous coronary intervention; CABG, coronary artery bypass grafting; CVI, cerebrovascular insult; CV, cardiovascular. Data are presented as median (IQR). Group comparisons were performed using the Mann–Whitney U test.

**Table 4:** Multiple linear regression model for predictors of Kauppila score.

Variable	B	SE	β	t	*p*-Value
Constant	−12.178	4.237		−2.874	0.005
Age	0.330	0.056	0.372	5.865	<0.001
Diabetes	3.808	1.022	0.224	3.727	<0.001
tGRP	0.002	0.001	0.135	2.235	0.027
eGFR	−0.062	0.016	−0.248	−3.833	<0.001

Abbreviations: B, unstandardized regression coefficient; SE, standard error of B; β, standardized regression coefficient; tGRP, total Gla-rich protein; eGFR, estimated glomerular filtration rate. Multiple linear regression included age, diabetes, tGRP, and eGFR as independent variables. Dependent variable: Kauppila score. Model statistics: F(4,180) = 36.471, *p* < 0.001; R^2^ = 0.394.

**Table 5:** Multiple linear regression model for predictors of PWV.

Variable	B	SE	β	t	*p*-Value
Constant	5.403	0.622	—	8.688	<0.001
Age	0.064	0.008	0.464	7.736	<0.001
Diabetes	0.281	0.150	0.107	1.874	0.063
tGRP	0.0002	0.0001	0.140	2.450	0.015
eGFR	−0.013	0.002	−0.325	−5.415	<0.001

Abbreviations: B, unstandardized regression coefficient; SE, standard error of B; β, standardized regression coefficient; tGRP, total Gla-rich protein; eGFR, estimated glomerular filtration rate; PWV, pulse wave velocity. Multiple linear regression included age, diabetes, tGRP, and eGFR as independent variables. Dependent variable: PWV. Model statistics: F(4,180) = 37.794, *p* < 0.001; R^2^ = 0.456.

**Table 6:** Multiple linear regression model for predictors of KLb.

Variable	B	SE	β	t	*p*-Value
Constant	4.777	2.754	—	1.735	0.087
eGFR	−0.053	0.043	−0.057	−1.235	0.221
tGRP	0.029	0.001	0.904	19.434	<0.001

Abbreviations: B, unstandardized regression coefficient; SE, standard error of B; β, standardized regression coefficient. Multiple linear regression models included eGFR and tGRP as independent variables. Dependent variable: KLb. Model statistics: F(2,78) = 204.30, *p* < 0.001; R^2^ = 0.839.

**Table 7:** Multiple linear regression model for predictors of FGF-23.

Variable	B	SE	β	t	*p*-Value
Constant	69.931	22.333	—	3.131	0.002
eGFR	−0.395	0.340	−0.062	−1.165	0.248
tGRP	0.210	0.013	0.875	16.558	<0.001

Abbreviations: B, unstandardized regression coefficient; SE, standard error of B; β, standardized regression coefficient. Multiple linear regression models included eGFR and tGRP as independent variables. Dependent variable: FGF-23. Model statistics: F(2,80) = 147.028, *p* < 0.001; R^2^ = 0.785.

## Data Availability

Anonymized data supporting the findings of this study are available from the corresponding author upon reasonable request.

## Supplementary Materials

The following supporting information can be downloaded at https://www.mdpi.com/article/10.3390/jcm15093374/s1, Table S1: Kolmogorov–Smirnov tests of normality for continuous variables; Table S2: Spearman correlation matrix of study variables; Table S3: Baseline characteristics according to availability of KLb and FGF-23 measurements.

## Abbreviations

The following abbreviations are used in this manuscript:

AO	Aortic valve calcification score
BMI	Body mass index
BP	Blood pressure
CABG	Coronary artery bypass grafting
Ca	Calcium
CKD	Chronic kidney disease
CKD-EPI	Chronic Kidney Disease Epidemiology Collaboration
CKD-MBD	Chronic kidney disease–mineral and bone disorder
CPP	Calciprotein particles
CV	Cardiovascular
CVD	Cardiovascular disease
CVI	Cerebrovascular insult
eGFR	Estimated glomerular filtration rate
ELISA	Enzyme-linked immunosorbent assay
ESRD	End-stage renal disease
EV	Extracellular vesicles
FGF-23	Fibroblast growth factor-23
GRP	Gla-rich protein
HD	Hemodialysis
IQR	Interquartile range
IVS	Interventricular septum thickness
KLb	β-Klotho
KS	Kolmogorov–Smirnov
LVH	Left ventricular hypertrophy
LVDd	Left ventricular end-diastolic diameter
LVPW	Left ventricular posterior wall thickness
MACE	Major adverse cardiovascular events
MGP	Matrix Gla protein
MI	Myocardial infarction
P	Phosphate
PCI	Percutaneous coronary intervention
PTH	Parathyroid hormone
PWV	Pulse wave velocity
T50	Serum calcification propensity test
tGRP	Total circulating GRP
ucGRP	Undercarboxylated Gla-rich protein
VC	Vascular calcification
VKDP	Vitamin K-dependent proteins
VSMC	Vascular smooth muscle cells
