# Supplementary material for: Gla-Rich Protein Across the Chronic Kidney Disease Spectrum: Association with Vascular Calcification Burden and CKD-MBD Disturbances
Source: J Clin Med. 2026 Apr 28;15(9):3374. doi: 10.3390/jcm15093374 (PMC13164490; doi:10.3390/jcm15093374)
Supplement: Supplementary file 1 [file jcm-15-03374-s001.zip › jcm-4236566-supplementary.pdf]

Supplementary Tables

Table S1. Kolmogorov–Smirnov tests of normality for continuous variables

Variable	D	df	p
Age (years)	0.058	185	0.200
eGFR	0.137	185	<0.001
Time to death from inclusion (months)	0.172	14	0.200
IVS (mm)	0.134	185	<0.001
LVPW (mm)	0.175	185	<0.001
AO calcification score	0.165	185	<0.001
LVDd (mm)	0.060	185	0.200
Systolic BP (mmHg)	0.048	185	0.200
Diastolic BP (mmHg)	0.094	185	<0.001
Pulse pressure (mmHg)	0.040	185	0.200
PWV (m/s)	0.102	185	<0.001
Height (cm)	0.048	185	0.200
Weight (kg)	0.090	185	0.001
BMI (kg/m^2)	0.109	185	<0.001
Kauppila score (0–24)	0.150	185	<0.001
β-Klotho (KLb) (pg/mL)	0.338	79	<0.001
tGRP (ng/L)	0.262	185	<0.001
FGF-23 (pg/mL)	0.266	81	<0.001
Calcium (mmol/L)	0.043	185	0.200
Phosphate (mmol/L)	0.112	185	<0.001
PTH (pmol/L)	0.194	185	<0.001
Hemodialysis duration (months)	0.236	63	<0.001

Abbreviations: eGFR, estimated glomerular filtration rate; IVS, interventricular septal thickness; LVPW, left ventricular posterior wall thickness; AO score, aortic calcification score; LVDd, left ventricular end-diastolic diameter; SBP/DBP, systolic/diastolic blood pressure; PWV, pulse wave velocity; BMI, body mass index; tGRP, total Gla-rich protein; FGF-23, fibroblast growth factor 23; PTH, parathyroid hormone. D = Kolmogorov–Smirnov statistic; df = degrees of freedom

Table S2. Spearman correlation matrix of study variables

Variable	1. Age	2. eGFR	3. IVS	4. LVPW	5. AO score	6. LVDd	7. SBP	8. DBP	9. PWV	10. BMI	11. Kaup-pila	12. KLb	13. tGRP	14. FGF-23	15. Ca	16. P	17. PTH
Age	1.00	-0.300*	0.171	0.132	0.458*	-0.090	-0.340*	0.088	0.568*	0.018	0.547*	-0.258	-0.059	-0.111	0.071	-0.090	0.139
eGFR		1.00	-0.411*	-0.409*	-0.535*	-0.195	-0.338*	0.097	-0.486*	-0.095	-0.429*	-0.344*	-0.220*	-0.291	0.351*	-0.391*	-0.669*
IVS			1.00	0.858*	0.452*	0.275*	0.176	0.021	0.198	0.270*	0.267*	-0.090	-0.025	-0.077	-0.139	0.142	0.233*
LVPW				1.00	0.445*	0.333*	0.170	0.016	0.212	0.204	0.249*	-0.014	0.036	-0.030	-0.196	0.120	0.262*
AO score					1.00	0.271*	0.371*	0.022	0.478*	0.122	0.533*	-0.041	0.100	-0.069	-0.168	0.143	0.332*
LVDd						1.00	0.159	-0.038	0.093	0.300*	0.075	-0.038	0.029	0.076	-0.292*	0.065	0.160
SBP							1.00	0.460*	0.666*	0.059	0.566*	0.020	0.117	0.190	-0.083	0.151	0.165
DBP								1.00	0.111	0.131	0.021	0.094	-0.044	0.004	-0.003	-0.142	-0.189
PWV									1.00	0.022	0.813*	0.073	0.149	0.200	-0.097	0.176	0.314*
BMI										1.00	0.102	-0.066	-0.104	-0.057	-0.077	0.099	0.008
Kauppila											1.00	-0.125	0.064	0.008	0.015	0.219*	0.221*
KLb												1.00	0.720*	0.673*	-0.192	0.221	0.307
tGRP													1.00	0.625*	-0.209	0.241*	0.185
FGF-23														1.00	-0.109	0.211	0.274
Ca															1.00	-0.211	-0.379*
P																1.00	0.330*
PTH																	1.00

Abbreviations: eGFR, estimated glomerular filtration rate; IVS, interventricular septal thickness; LVPW, left ventricular posterior wall thickness; AO score, aortic calcification score; LVDd, left ventricular end-diastolic diameter; SBP/DBP, systolic/diastolic blood pressure; PWV, pulse wave velocity; BMI, body mass index; tGRP, total Gla-rich protein; FGF-23, fibroblast growth factor 23; PTH, parathyroid hormone.

Table S3A. Baseline characteristics according to availability of KLb measurements

Variable	KLb unavailable (n = 106)	KLb available (n = 79)	Test statistic	p-value
Age, years	67 (62–73)	67 (61–75)	U = 4049.50	0.702
Male sex, n (%)	45 (42.5)	46 (58.2)	χ^2 = 4.507	0.034
Diabetes, n (%)	44 (41.5)	34 (43.0)	χ^2 = 0.043	0.836
Hypertension, n (%)	87 (82.1)	71 (89.9)	χ^2 = 2.208	0.137
eGFR, mL/min/1.73 m^2	36.0 (10.75–78.25)	23.0 (6.0–42.0)	U = 3254.00	0.010
Kauppila score	7 (2.75–15)	12 (3–20)	U = 3523.00	0.065
PWV, m/s	9.6 (8.57–10.5)	9.8 (8.4–10.7)	U = 3740.50	0.215
BMI, kg/m^2	28.0 (26.0–30.1)	29.1 (25.5–32.9)	U = 3796.00	0.278

Abbreviations: KLb, β-Klotho; eGFR, estimated glomerular filtration rate; PWV, pulse wave velocity; BMI, body mass index. Data are presented as median (IQR) for continuous variables and n (%) for categorical variables. Continuous variables were compared using the Mann–Whitney U test, and categorical variables using the chi-square test.

Table S3B. Baseline characteristics according to availability of FGF-23 measurements

Variable	FGF-23 unavailable (n = 104)	FGF-23 available (n = 81)	Test statistic	p-value
Age, years	68 (62–73)	67 (61–74)	U = 4194.00	0.960
Male sex, n (%)	44 (42.3)	47 (58.0)	χ^2 = 4.500	0.034
Diabetes, n (%)	45 (43.3)	33 (40.7)	χ^2 = 0.119	0.730
Hypertension, n (%)	85 (81.7)	73 (90.1)	χ^2 = 2.208	0.137
eGFR, mL/min/1.73 m^2	33.5 (10–78)	26.0 (6–44)	U = 3485.00	0.044
Kauppila score	8 (3–15)	11 (2.5–20)	U = 3816.00	0.272
PWV, m/s	9.65 (8.6–10.5)	9.8 (8.35–10.7)	U = 3953.50	0.215
BMI, kg/m^2	27.95 (25.92–29.95)	29.3 (25.7–33.15)	U = 3657.00	0.125

Abbreviations: FGF-23, fibroblast growth factor 23; eGFR, estimated glomerular filtration rate; PWV, pulse wave velocity; BMI, body mass index. Data are presented as median (IQR) for continuous variables and n (%) for categorical variables. Continuous variables were compared using the Mann–Whitney U test, and categorical variables using the chi-square test.
